# Diabetes Mellitus in Neonates and Infants: Genetic Heterogeneity, Clinical Approach to Diagnosis, and Therapeutic Options

**DOI:** 10.1159/000354219

**Published:** 2013-09-18

**Authors:** Oscar Rubio-Cabezas, Sian Ellard

**Affiliations:** ^a^Department of Paediatric Endocrinology, Hospital Infantil Universitario Niño Jesús, Madrid, Spain; ^b^Institute of Biomedical and Clinical Science, University of Exeter Medical School, Exeter, UK

**Keywords:** Neonatal diabetes, Monogenic diabetes of infancy, Permanent neonatal diabetes, Transient neonatal diabetes, Type 1 diabetes

## Abstract

Over the last decade, we have witnessed major advances in the understanding of the molecular basis of neonatal and infancy-onset diabetes. It is now widely accepted that diabetes presenting before 6 months of age is unlikely to be autoimmune type 1 diabetes. The vast majority of such patients will have a monogenic disorder responsible for the disease and, in some of them, also for a number of other associated extrapancreatic clinical features. Reaching a molecular diagnosis will have immediate clinical consequences for about half of affected patients, as identification of a mutation in either of the two genes encoding the ATP-sensitive potassium channel allows switching from insulin injections to oral sulphonylureas. It also facilitates genetic counselling within the affected families and predicts clinical prognosis. Importantly, monogenic diabetes seems not to be limited to the first 6 months but extends to some extent into the second half of the first year of life, when type 1 diabetes is the more common cause of diabetes. From a scientific perspective, the identification of novel genetic aetiologies has provided important new knowledge regarding the development and function of the human pancreas.

Presentation of diabetes within the first year of life was first reported in 1789 [1]. The fact that most cases occurred predominantly in the first and last quarters of the first year, suggesting some aetiologic heterogeneity in these patients, was recognized over 50 years ago [2] but did not receive adequate attention until recently [3]. The rarity of the condition, with an overall estimated incidence of less than 2 cases per 100,000 infants [4,5], might have played a role in this lack of awareness. However, the enormous amount of novel information gathered since the publication of the human genome a decade ago has increased our understanding of the pathophysiology of this rare condition and had a huge impact on routine clinical care for diabetic infants.

## Diabetes Mellitus in Infants: The Basics

Only a few years ago, type 1 diabetes (T1D) was considered the main form of diabetes presenting in infancy, and the term ‘neonatal diabetes’ (NDM) was reserved for diabetes presenting within the first 4-6 weeks of life [6].

### Type 1 Diabetes

**Fig. 1:**
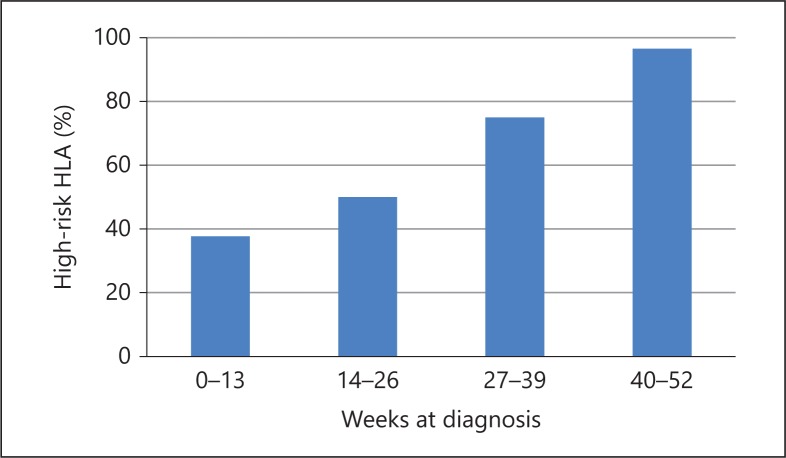
HLA distribution in infants diagnosed with diabetes mellitus under 1 year of age. Modified from Edghill et al. [25].

T1D is a T cell-mediated autoimmune disease that results from a selective destruction of the pancreatic insulin-producing β-cells and is the most common cause of diabetes in children, accounting for over 95% of cases [7]. The disease results from a combination of genetic predisposition and a number of potential environmental factors. Genome-wide association studies have identified over 40 loci contributing to T1D [8], but allelic variation within the HLA class II region in the major histocompatibility complex on chromosome 6p21.3 contributes about 50% of the inherited risk [9]. The earlier the clinical onset of the disease, the stronger the genetic susceptibility, especially when diabetes presents before 2 years of age [10]. However, when the HLA haplotype distribution was studied in infants diagnosed with diabetes within the first year of life, the distribution in infants presenting at or after 7 months was similar to older T1D patients (95% positive for high-risk HLA), whereas younger patients diagnosed before 6 months were similar to control subjects (40% positive; fig. 1) [3,11].

The environmental triggers that initiate pancreatic β-cell destruction in T1D remain largely unknown. Pancreatic autoantibodies are a hallmark of the underlying autoimmune process and their presence long precedes clinical diagnosis of T1D [12,13], but prospective studies in newborns with HLA-defined genetic high risk for T1D have shown that autoimmunity does not usually evolve rapidly enough to cause clinical T1D in the first months of life [14,15,16]. In keeping with this, infants diagnosed with diabetes before 6 months of age were less likely to be antibody positive than infants diagnosed later (15 vs. 65%) [3]. Even though autoimmune diabetes is rare in very young infants [3], it is now accepted that *FOXP3* mutations, and not T1D, will account for most of these cases [17].

Almost two thirds of the infants diagnosed in the first 6 months were born small for gestational age, in contrast to 15% of those presenting between 6 and 12 months [3]. On the other hand, a recent meta-analysis has shown that higher birthweights are associated with a mildly increased risk of developing T1D [18]. Since insulin exerts potent growth-promoting effects during intrauterine development [19], a low birthweight in infants diagnosed with diabetes soon after birth might reflect reduced insulin secretion by the foetal pancreas due to β-cell dysfunction in utero.

### Monogenic, Non-Type 1 Diabetes

According to the information displayed above, clinical presentation of polygenic autoimmune T1D within the first 6 months of life is rare. The majority of these patients will have a monogenic form of diabetes [20].

Over the last few years, the age limit to consider a non-autoimmune, monogenic cause has changed from the first 30-45 days of life [6] to 3 months, then 6 months [21] and now 9 months [22]. Monogenic diabetes of infancy has been suggested to be a more appropriate name than NDM [21], but the latter is still extensively used and preferred by many authors. Approximately 50% of the NDM patients will require lifelong treatment to control hyperglycaemia (permanent NDM, PNDM). In the remaining patients, diabetes will remit within a few weeks or months (transient NDM, TNDM), although it might relapse later in life. In both cases, diabetes is more frequently isolated, but some cases show a variety of associated extra-pancreatic clinical features pointing to a particular gene, which may help guide genetic testing (table 1) [20].

## Genetic Heterogeneity in Infancy-Onset Monogenic Diabetes

NDM is genetically heterogeneous, with at least 20 different causal genes identified to date (table 1). The vast majority of the monogenic causes of NDM result in impaired insulin secretion rather than insulin sensitivity [23]. From an aetiopathogenic perspective, insulin deficiency appears as a result of one of three alternative mechanisms: either impaired development or function of β-cells, and progressive β-cell destruction (table 1). Whilst the genetic basis of TNDM has been mostly uncovered, with three genetic abnormalities accounting for the majority of patients [24], the gene mutated in up to 40% of PNDM cases remains unknown [25].

### Permanent Neonatal Diabetes

**Fig. 2:**
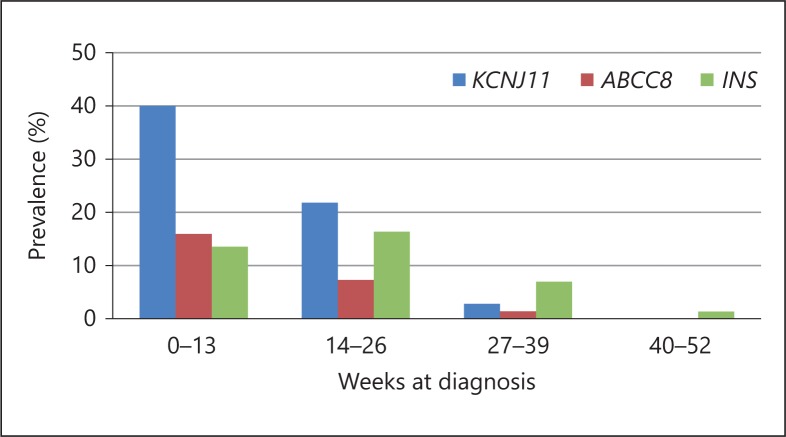
Prevalence of the three more common genetic subtypes of monogenic diabetes during the first year of life. Redrawn from Rubio-Cabezas et al. [22].

Activating mutations in either the *KCNJ11* or *ABCC8* genes encoding the two subunits (Kir6.2 and SUR1, respectively) of the ATP-sensitive potassium (K_ATP_) channel of the β-cell membrane, prevent insulin secretion in response to hyperglycaemia and can cause both PNDM and TNDM [26,27,28]. *KCNJ11* mutations are more frequent, and most patients have PNDM rather than TNDM. In contrast, mutations in *ABCC8* cause TNDM more frequently. Consistent with the expression of K_ATP_ channels in neurons [29], about 20% of probands with *KCNJ11* mutations present with associated neurological symptoms [30]. The most severe defect includes marked developmental delay and early-onset epilepsy, and has been called DEND syndrome. An intermediate DEND syndrome characterized by NDM and less severe developmental delay without epilepsy is more common. Neurological features can also present in patients with mutations in *ABCC8* but are less frequent and usually milder (language delay and dyspraxia, mainly) [28,31]. There are no significant clinical differences between these two subtypes of monogenic diabetes regarding the severity of intrauterine growth retardation (median birthweight around −1.5 SDS for gestational age) or the median age at diabetes presentation (4-8 weeks) [24,25]. Diabetes is diagnosed within the first 6 months in the vast majority of patients, although they can occasionally present beyond 6 months [22] (see fig. 2). More than 90% of patients with activating mutations in the K_ATP_ channel genes can transfer from insulin to high-dose sulphonylureas, with improved glycaemic control and decreased risk of hypoglycaemia [32,33,34]. Preliminary data also suggest that glibenclamide might partially improve or even prevent some of the associated neurological symptoms [35,36,37].

Heterozygous coding mutations in the proinsulin gene *(INS)* are the second most common cause of isolated PNDM [25,38,39,40]. The mutation usually results in accumulation of a misfolded proinsulin molecule in the endoplasmic reticulum (ER), leading to ER stress and β-cell apoptosis [40]. The severity of the intrauterine growth retardation in patients with *INS* mutations is similar to that of patients with K_ATP_ channel mutations, but diabetes tends to present slightly later [25], even in late infancy or childhood [22,39,41]. Since there is a progressive β-cell death, insulin is the only treatment currently available.

**Fig. 3:**
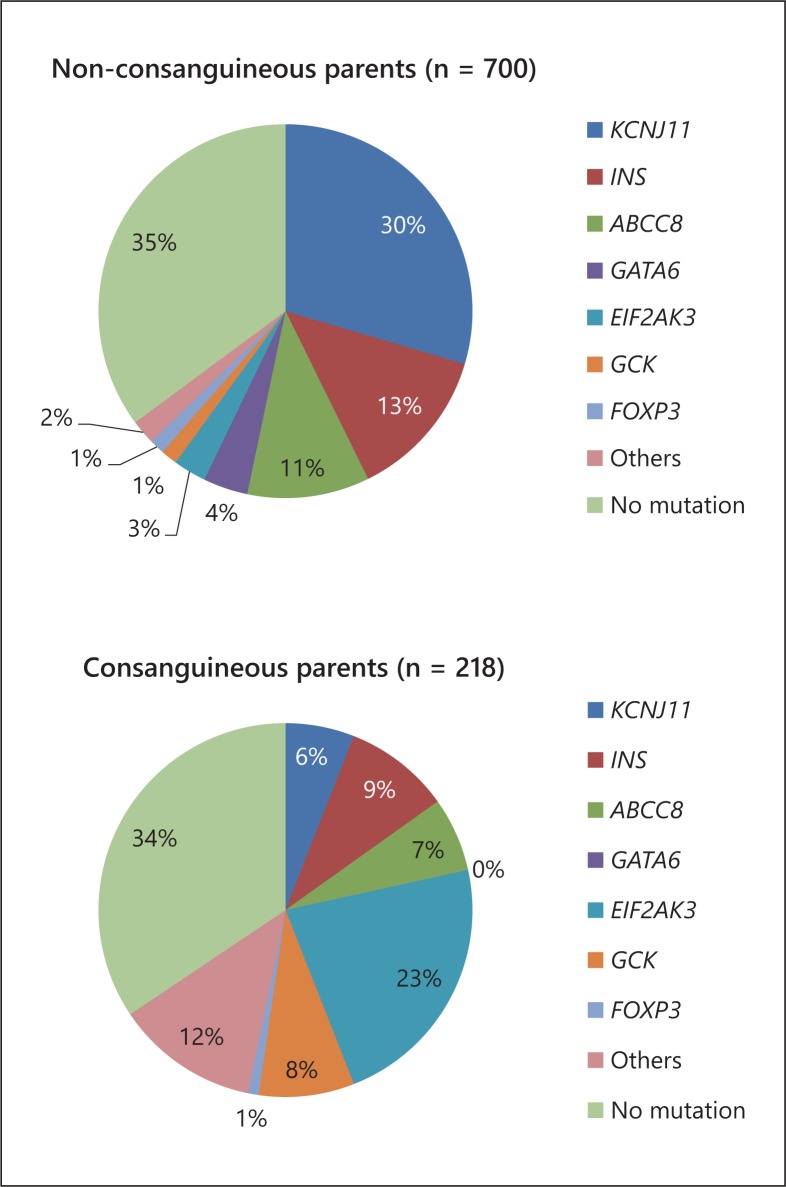
Influence of parental consanguinity on the genetic aetiology in the Exeter cohort of PNDM. Updated from Rubio-Cabezas et al. [72].

Mutations in the K_ATP_ channel genes and the proinsulin gene *(INS)* account for at least 50-75% of infancy-onset permanent monogenic diabetes in outbred populations [22,25,42,43]. However, parental consanguinity exerts a strong influence on both the prevalence and the genetic aetiology of NDM, with mutations in *EIF2AK3* being the commonest known genetic cause (fig. 3) [44,45]. All mutations reported to date in *KCNJ11* and most mutations in *ABCC8* and *INS* are heterozygous and, hence, dominantly acting. These mutations frequently appear de novo, and consequently there is no positive family history of diabetes suggesting a genetic disorder. Therefore, these patients cannot be clearly distinguished from those with early-onset T1D just on clinical grounds. In contrast, some mutations in *ABCC8* and *INS*, and all mutations in *EIF2AK3* -causing Wolcott-Rallison syndrome-, *GCK*, and other genes less frequently involved in NDM are homozygous or compound heterozygous, indicating a recessive inheritance. The risk of developing one of these recessive subtypes of NDM is therefore increased when parental consanguinity is present. However, K_ATP_ channel mutations account for a minority of consanguineous cases, indicating that the likelihood of transitioning from insulin onto oral sulphonylureas is significantly lower when parents are related.

### Transient Neonatal Diabetes

The majority of cases (∼70%) are linked to 3 different abnormalities in an imprinted region on chromosome 6q24 ultimately leading to overexpression of a *PLAGL1*: paternal duplication, paternal uniparental disomy, and abnormal methylation of the maternal allele [46,47]. The latter methylation defect sometimes arises in the context of a generalized hypomethylation syndrome [48] secondary to biallelic mutations in *ZFP57*, a gene involved in the regulation of DNA methylation [49].

Patients with 6q24 abnormalities are born with moderate intrauterine growth retardation (average birthweight: 1,930 g) and usually develop severe non-ketotic hyperglycaemia during the first week of life [47,50]. Despite the severity of the initial presentation, diabetes remits in the majority of patients by a median age of 12 weeks. During remission, transient hyperglycaemia may occur during intercurrent illnesses [51]. Diabetes tends to relapse around puberty but may do so from the age of 4 years. Clinically, relapse resembles early-onset type 2 diabetes. Insulin therapy is not always necessary as diabetes may respond to oral sulphonylureas but, if needed, insulin doses required are lower than in patients with T1D.

Genetic counselling for families with 6q24 TNDM depends on the underlying molecular mechanism. Uniparental disomy of chromosome 6 is generally sporadic and therefore the risk of recurrence in siblings and offspring is low. For paternal duplication of the 6q24 region, males have a 50% chance of transmitting the mutation and the disease to their children. In contrast, females will pass on the duplication, but their children will not develop the disease, which may recur instead in the next generation as their asymptomatic sons pass on the molecular defect to their own children. Methylation defects are usually sporadic, but mutations in *ZFP57* show an autosomal recessive inheritance, and hence the recurrence risk is 25% for siblings and almost negligible for the offspring of an affected patient.

Activating mutations in any of the genes (*KCNJ11* and *ABCC8*) encoding the two subunits (Kir6.2 and SUR1, respectively) of the K_ATP_ channel of the β-cell membrane account for most of the remaining cases of TNDM [27,28]. Compared to patients with 6q24 abnormalities, TNDM patients with activating K_ATP_ channel mutations show milder intrauterine growth retardation and are diagnosed later, suggesting that the prenatal insulin deficiency is less severe. In addition, diabetes tends to remit later and relapses sooner [24].

### Recent Identification of New Genetic Subtypes

Since NDM was last reviewed in this journal in 2007 [52], a number of new genes underlying this diagnosis have been identified. The most relevant one from a clinical perspective was undoubtedly *INS*, the gene encoding insulin [38], which accounts for about 10-15% of NDM cases and is the second most common cause after activating mutations in the K_ATP_ channel genes [25,39,40,42]. Since *INS* is mostly expressed in pancreatic β-cells, the affected patients do not show any extrapancreatic features. In addition to causing NDM, *INS* mutations may present acutely after the first 6 months of age and even beyond the age of one year, when monogenic diabetes becomes exceedingly rare [41]. Furthermore, up to 70% of the mutations are de novo, so that family history of early-onset diabetes is lacking. It is therefore likely that some patients with an *INS* mutation are clinically indistinguishable from early-onset T1D. In these cases, routine measurement of pancreatic autoantibodies in young children with diabetes may be a helpful tool to identify candidates for genetic testing [53].

A second area where major advances have recently been achieved is syndromic, early-onset diabetes, i.e. diabetes associated with varied extrapancreatic features, which represents about 10% of cases. Most of the newly identified genetic causes are transcription factors important for pancreatic development at different stages (table 1). Many were suggested as candidate genes from mouse knockout models. The first novel aetiology to be identified by next generation sequencing technology was *GATA6* haploinsufficiency [54]. De novo mutations were identified by exome sequencing of affected proband/unaffected, unrelated parent trios before Sanger sequencing in a wider cohort showed that heterozygous *GATA6* mutations are the most common cause of pancreatic agenesis [54]. The phenotypic spectrum has recently been extended with the description of *GATA6* mutations in patients with PNDM, TNDM or adult-onset diabetes [55]. Cardiac malformations are present in most cases.

When the mutated transcription factor is normally expressed early in development (for instance, *RFX6* or *GATA6*), both endocrine and exocrine lineages are involved, and the affected patient shows some degree of pancreatic hypoplasia and exocrine dysfunction, in addition to the associated extrapancreatic features secondary to the extrapancreatic expression of the respective gene [54,55,56]. On the contrary, when the expression of the transcription factor is limited to the endocrine pancreas (i.e. *NEUROG3*, *NEUROD1*, *PAX6*), pancreatic hypoplasia and exocrine dysfunction are lacking [57,58,59]. Importantly, the presence of extrapancreatic features does not necessarily indicate a developmental disorder. This is exemplified by mutations in *IER3IP1*, which cause NDM plus lissencephaly and other neurological abnormalities by inducing ER-associated apoptosis [60].

Despite the above-referenced recent advances, up to 40% of patients with NDM remain without a molecular genetic diagnosis [25], most of them not showing any associated extrapancreatic clinical manifestations, which suggests that other genes are yet to be discovered.

## Clinical Approach to Genetic Testing in Infancy-Onset Diabetes

Reaching a specific molecular diagnosis in patients with suspected monogenic diabetes has important clinical consequences as it may influence diabetes treatment and define the prognosis in the affected subject as well as in other family members. Whilst genetic testing confirms or excludes a diagnosis of monogenic diabetes with both high sensitivity and high specificity, molecular studies are expensive and time-consuming, and some criteria must be used to select candidates for genetic testing.

Age at diabetes onset can be considered the cornerstone criterion since most infants diagnosed before 6 months will have monogenic diabetes [20]. It has recently been demonstrated that genetic testing for mutations in the K_ATP_ channel genes in these patients is cost-effective [61] and therefore both genes should be screened in the first place. If no mutations are identified, *INS* is the next gene to investigate. Testing for 6q24 abnormalities should be considered in case where diabetes has already remitted at the time of referral or if the patient was born with significant low birthweight, is still aged <3-6 months (before possible remission), and diabetes presented within the first week of life. If this is also negative, further testing may be guided by additional clinical criteria.

The history and physical examination, either of the patient or other affected family members, are the best and cheapest tools available for this purpose [62]. Associated extra-pancreatic clinical features might point to specific genes, most of which (apart from *HNF1B* and *GATA6*), cause autosomal recessive disorders (as shown in table 1). Parental consanguinity also suggests an autosomal recessive syndrome. However, absence of known consanguinity does not exclude autosomal recessive inheritance, especially in populations with a high consanguinity rate.

Patients presenting with diabetes during the first 6 months of life tend to be small for gestational age at birth [3,63]. However, the degree of intrauterine growth retardation among patients with different subtypes of NDM varies widely. Patients unable to produce any insulin both prenatally and postnatally due to pancreatic agenesis or biallelic mutations in *GCK* or *INS* show lower birthweights, usually below −3 SDS for gestational age [64,65,66], compared to patients with heterozygous *INS* mutations or Wolcott-Rallison syndrome where birthweight, although typically low, is usually above −2 SDS for gestational age [25,44]. This is in keeping with the underlying mechanism causing NDM, with the latter two examples involving progressive destruction of normally developed β-cells and therefore prenatal insulin secretion being relatively spared.

Some laboratory tests may also prove useful. The size of the pancreas and the exocrine function may be evaluated by a number of imaging and biochemical tests – abdominal US, CT or MRI scans, and faecal fat or elastase, respectively. In selected patients, other tests should be considered including, but not limited to, liver and kidney function tests, X-ray bone survey, audiogram, echocardiogram and/or brain MRI. Positive autoantibodies to β-cells may indicate IPEX syndrome when identified in a male patient presenting with NDM soon after birth or, alternatively, suggest very early-onset T1D in a patient (male or female) who developed diabetes slightly before 6 months of age.

It is important to keep in mind that some infants presenting with diabetes after 6 months will also have monogenic diabetes, especially when pancreatic autoantibodies are negative. If there are no associated clinical features, screening for *INS* mutations followed by K_ATP_ channel genes testing should be considered. Apart from Wolcott-Rallison syndrome, late infancy-onset diabetes is exceedingly rare in other forms of syndromic monogenic diabetes.

The advent of next-generation sequencing technologies brings the possibility of simultaneous testing for all known NDM genes in a single assay [67], and this increases the diagnostic rate for patients with NDM.

## Options for Treatment and Management

Early insulin administration is acutely required in most infants with newly diagnosed diabetes to treat or prevent acute metabolic decompensation and to allow weight gain [68]. Caloric and glucose restriction in an attempt to avoid or delay insulin therapy should be strongly discouraged. Insulin treatment in infants is always challenging and must be carefully designed in keeping with their nutritional management and frequent glucose monitoring.

Insulin can be provided by multiple daily injections or continuous subcutaneous infusion [69]. It may be necessary to use diluted insulin to meet very low insulin requirements and minimize the risk of hypoglycaemia. In infants with parenteral nutrition or continuous enteral feeding, the total daily dose of insulin should be given as basal infusion. As in healthy infants, breastfeeding is recommended, and its frequency influences insulin requirements. With more than 6 breast feeds per day, high basal insulin substitution with very low mealtime boluses allows for a stable blood glucose levels. When more intermittent oral feeding is started, increased insulin requirements associated to meals require increased doses of rapid-acting insulin analogues and the basal insulin has to be reduced accordingly, to 30-50% of total daily dose in infants with multiple daily injections or to 10-30% with continuous subcutaneous infusion. Since food intake in infants is frequently unpredictable, immediate postprandial insulin administration might be considered in certain circumstances. Extra-slowly-absorbed complex carbohydrates such as corn starch may be given at bedtime to prevent nocturnal hypoglycaemia.

Most patients with activating mutations in *KCNJ11* or *ABCC8* can be successfully switched from insulin to oral sulphonylurea [32,33], with a marked improvement in metabolic control. Transfer protocols are available at www.diabetegenes.org. The required doses of sulphonylurea are usually high, especially in patients with neurological features, but tend to decrease over time irrespective of the mutation. Long-term follow-up has revealed persistent efficacy and safety [34]. The only side effects reported to date are transient diarrhoea at transfer [70] and staining of the teeth in the longer term [71].

Patients with agenesis of the pancreas require pancreatic enzyme supplements in addition to insulin treatment.

## Conclusions

When a newborn or infant presents with diabetes, both clinicians and families face a highly complex situation. Reaching a molecular diagnosis confirms the genetic subtype, predicts the prognosis and possible development of extra-pancreatic features, and determines the risk of diabetes in future siblings/offspring. The most dramatic impact is for those patients with a K_ATP_ channel mutation who are able to transfer from insulin injections to sulphonylurea tablets and achieve improved glycaemic control. More than 20 different causal genes have now been identified, with many new aetiologies discovered through a combination of homozygosity/sequencing analysis of candidate genes suggested from mouse knockout studies. Exome sequencing has revealed *GATA6* haploinsufficiency as the most common cause of pancreatic agenesis, and the expectation is that genome-wide analysis by next generation sequencing will rapidly uncover additional new causal genes to assist in the diagnosis and clinical management of NDM.

## Figures and Tables

**Table 1:** Monogenic subtypes of neonatal and infancy-onset diabetes mellitus

Gene	Locus	Inheritance	Other clinical features
*Abnormal pancreatic development*			
*PLAGL1*	6q24	variable (imprinting)	TNDM ± macroglossia ± umbilical hernia

*ZFP57*	6p22.1	recessive	TNDM (multiple hypomethylation syndrome) ± macroglossia ± developmental delay ± umbilical defects ± congenital heart disease

*PDX1*	13q12.1	recessive	PNDM + pancreatic agenesis (steatorrhoea)

*PTF1A*	10p12.3	recessive	PNDM + pancreatic agenesis (steatorrhoea) + cerebellar hypoplasia/aplasia + central respiratory dysfunction

*HNF1B*	17cen-q21.3	dominant	TNDM + pancreatic hypoplasia and renal cysts

*RFX6*	6q22.1	recessive	PNDM + intestinal atresia + gall bladder agenesis

*GATA6*	18q11.1-q11.2	dominant	PNDM + congenital heart defects + biliary abnormalities

*GLIS3*	9p24.3-p23	recessive	PNDM + congenital hypothyroidism + glaucoma + hepatic fibrosis + renal cysts

*NEUROG3*	10q21.3	recessive	PNDM + enteric anendocrinosis (malabsorptive diarrhoea)

*NEUROD1*	2q32	recessive	PNDM + cerebellar hypoplasia + visual impairment + deafness

*PAX6*	11p13	recessive	PNDM + microphthalmia + brain malformations

*Abnormal β-cell function*			
*KCNJ11*	11p15.1	spontaneous or dominant	PNDM/TNDM ± DEND

*ABCC8*	11p15.1	spontaneous, dominant or recessive	TNDM/PNDM ± DEND

*INS*	11p15.1	recessive	Isolated PNDM or TNDM

*GCK*	7p15-p13	recessive	Isolated PNDM

*SLC2A2* (GLUT2)	3q26.1-q26.3	recessive	Fanconi-Bickel syndrome: PNDM + hypergalactosemia, liver dysfunction

*SLC19A2*	1q23.3	recessive	Roger's syndrome: PNDM + thiamine-responsive megaloblastic anaemia, sensorineural deafness
*Destruction of β-cells*		

*INS*	11p15.1	spontaneous or dominant	Isolated PNDM
*EIF2AK3*	2p12	recessive	Wolcott-Rallison syndrome: PNDM + skeletal dysplasia + recurrent liver dysfunction

*IER3IP1*	18q12	recessive	PNDM + microcephaly + lissencephaly + epileptic encephalopathy

*FOXP3*	Xp11.23-p13.3	X-linked, recessive	IPEX syndrome (autoimmune enteropathy, eczema, autoimmune hypothyroidism, elevated IgE)

*WFS1*	4p16.1	recessive	PNDM + optic atrophy ± diabetes insipidus ± deafness
